# Hydrodissection in microwave ablation: the effectiveness of 0.9 % NaCl versus 5 % dextrose in an ex vivo experimental set-up

**DOI:** 10.1016/j.redii.2025.100060

**Published:** 2025-05-20

**Authors:** Gonnie C.M. van Erp, Pim Hendriks, Sophie A. van den Hurk, Hannah F. Winder, Willemijn P.M. Scholtes, Lara B.E.M. De Bats, Jouke Dijkstra, Mark C. Burgmans

**Affiliations:** a Interventional Radiology Research (IR2) Group, Department of Radiology, Leiden University Medical Center, 2300 RC, Albinsudreef 2, Leiden 2333 ZA, the Netherlands; b Division of Image Processing, Department of Radiology, Leiden University Medical Center, 2300 RC, Leiden, the Netherlands

**Keywords:** Thermal ablation, Microwave ablation, Hydrodissection, 0.9 % NaCl, D5W

## Abstract

**Purpose:**

To compare the effectiveness of hydrodissection using 0.9 % NaCl (saline) or 5 % dextrose in water during microwave ablation at different hydrodissection fluid thicknesses, in an ex vivo experimental set-up.

**Methods:**

Two porcine liver parts were placed in a plastic container simulating a superficial liver ablation with adjacent tissue. The space between the livers was filled with either saline or 5 % dextrose in water. Microwave ablation was performed 4 min at 100 W, at 15 mm from the liver surface. Three thermocouples were used to determine the heat propagation: (1) between the microwave ablation antenna and liver surface; (2) 5 mm from the surface of the adjacent tissue; (3) 15 mm from the surface of the adjacent tissue. Forty experiments were performed using hydrodissection fluid thicknesses ranging from 1 to 10 mm. The maximum temperature increase for each thermocouple was determined. A Spearman’s correlation analysis assessed the relationship between the hydrodissection fluid thickness (in millimeters) and the temperature increase (in degrees Celsius) per fluid.

**Results:**

At 5 mm within the adjacent tissue, use of 1 mm hydrodissection fluid thickness with 5 % dextrose in water resulted in less temperature increase (4.6 °C) compared to saline (6.8 °C). Additionally, at this distance, a negative correlation was observed between hydrodissection fluid thickness and temperature increase for both saline hydrodissection (*r*(18) = −0.96, *p* < 0.001) and 5 % dextrose in water hydrodissection (*r*(18) = -0.81, *p* < 0.001), which differs significantly (*p* = 0.011).

**Conclusion:**

Results from this experimental ex vivo study suggest that 5 % dextrose in water may protect adjacent critical structures better from heating during microwave ablation than saline.

## 1 Introduction

Thermal ablation is a first line therapy for both primary and secondary liver malignancies. Major complications after thermal liver ablation are rare with a reported incidence of 2.2–3.1 % [1]. One of the most feared complications is thermal damage to adjacent structures, such as the diaphragm, intestines, stomach, and gallbladder. The severity of such thermal injuries varies from pain to organ perforation. Organ perforation may have grave consequences, such as diaphragmatic hernia, enterocutaneous fistula formation, peritonitis, septic shock, and even death [[2], [3], [4]].

To protect adjacent structures from thermal damage, hydrodissection may be applied during thermal ablation [5,6]. Hydrodissection involves the insertion of a thin needle between the liver and the adjacent tissue through which a fluid is infused. The protection of hydrodissection is twofold. Firstly, hydrodissection creates space between the liver and the adjacent tissue. Secondly, the fluid acts as an insulator, limiting the transfer of heat to the adjacent tissue. In addition to providing protection, hydrodissection helps to improve the primary technique efficacy of hepatic superficial tumor ablation [7]. Hydrodissection provides radiologists with greater confidence to ablate the tumor more aggressively, while minimizing the complication risk. Lesions that were previously considered untreatable may now be successfully ablated.

Different fluids may be used for hydrodissection, with 5 % dextrose in water (D5W) solution and 0.9 % NaCL solution in water (saline) being most frequently used. In radiofrequency ablation (RFA), hydrodissection using D5W is most effective [[6], [7], [8]]. RFA applies a rapidly alternating electric current resulting in ionic agitation and subsequent heating of tissue. An ionic fluid, such as saline, can propagate the electric current into adjacent tissue and should thus be avoided when applying RFA. D5W is an isoosmolar and non-ionic fluid with low electrical conductivity providing both a physical and electrical barrier in RFA. However, the use of D5W also poses a risk of electrolyte imbalance in patients, if used in large volumes [9].

RFA has been the most used thermal ablation technique for years, but over recent years many centers have adopted microwave ablation (MWA) as their preferred technique. MWA uses dielectric hysteresis to produce direct tissue heating and is not dependent on ionic agitation [10,11]. Therefore, there is less rationale to use D5W for hydrodissection in MWA. Saline, which is widely available and well tolerated by the body, might offer the same insulating effect in MWA, without posing the risk of electrolyte imbalance. Currently, hydrodissection techniques have mostly been adopted from those used for RFA. Little is known on what the optimal hydrodissection technique is for MWA. Therefore, this experimental ex vivo study compares the effectiveness of hydrodissection using saline or D5W during microwave ablation at different hydrodissection fluid thicknesses.

## 2 Materials and methods

### 2.1 Study design

An experimental ex vivo study was performed using 9.5 kg of porcine livers collected from an abattoir. The livers were cut in parts measuring 60 mm in width and 50 mm in height with a thickness varying between 20 and 70 mm, depending on the thickness of the liver. The livers were stored in a freezer and defrosted at room-temperature at least 12 h before the experiments. The experiments were performed at room-temperature.

### 2.2 Experimental set-up

**Fig. 1:**
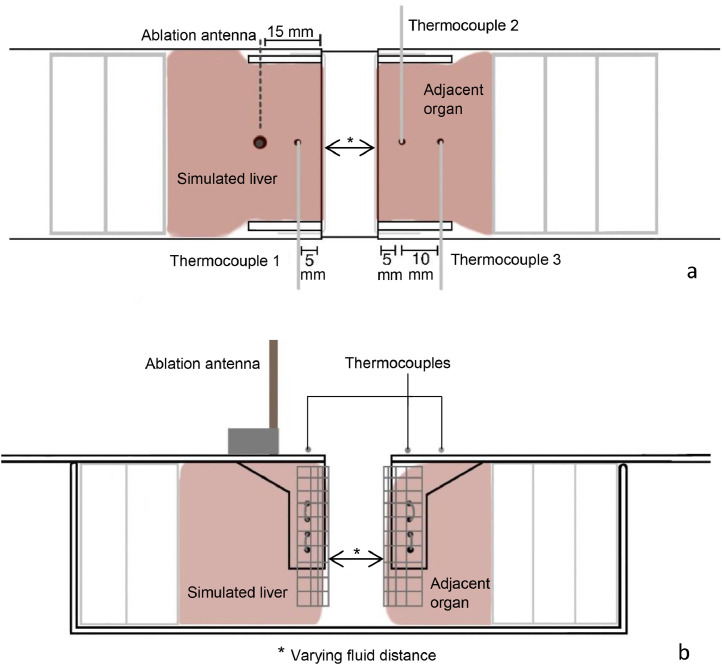
Schematic overview of the top view (a) and sideview (b) of the ex vivo porcine liver set up used for the experiments.

Fig. 1 presents a schematic top and side view of the test set-up. Two porcine liver parts were placed in a plastic container with one part simulating a superficial liver ablation and the other part simulating adjacent tissue. The liver parts were stabilized with a metal mesh sheet. The space between the two liver parts varied over the experiments and was filled with hydrodissection fluid, either saline or D5W. Two laser-cut plexiglass plates, with openings guiding precise insertion for the ablation antenna and thermocouples, were put on top of the box. These plexiglass templates ensured a controlled test set-up with fixed positions of the ablation antenna and thermocouples in all experiments.

The Emprint HP (Medtronic, Mansfield USA) microwave system was used in all experiments with an ablation time of 4 min and a power of 100 W. The MWA antenna was inserted 15 mm from the liver surface at a fixed depth of 38.5 mm. Moreover, three thermocouples (Thermocouple type K, RS PRO, The Netherlands) were inserted: thermocouple 1 at 5 mm from the liver surface in between the MWA antenna and the liver surface, thermocouple 2 in the adjacent tissue at 5 mm from the liver surface and thermocouple 3 at 15 mm of the surface of the adjacent tissue. All of the thermocouples were positioned at a fixed depth of 23 mm to align them with the center of the ablation zone.

The temperature measurements started 20 s before the start of the ablation and continued for at least 60 s after the end of the ablation. Every second, the temperature of all three thermocouples were recorded in Laboratory Virtual Instrumentation Engineering Workbench (LabVIEW, version 2018, National Instruments) software.

Ten different hydrodissection fluid thicknesses, ranging from 1 mm to 10 mm, were used. Experiments were performed for both D5W and saline at each hydrodissection fluid thickness twice, resulting in 40 intended experiments.

### 2.3 Statistical analyses

Statistical analyses were performed in R Statistical Software (version 4.3.1). Temperatures are reported as median with interquartile range. The maximum temperature increase in the adjacent tissue was determined for each experiment and plotted against the hydrodissection fluid thickness, for each fluid separately. A Spearman’s rank-order correlation was computed to assess the relationship between the hydrodissection fluid thickness (in millimeters) and the temperature increase (in degrees Celsius) per fluid. The differences between the correlation coefficients of the fluids were assessed using the Fisher's z transformation. The level of statistical significance was set at 0.05.

## 3 Results

A total of 41 experiments were performed, of which one was excluded after premature termination of ablation due to dislocation of thermocouple 1 during the experiment. Consequently, 40 experiments were included for analysis.

Table 1 gives the descriptive statistics of the temperature measured in thermocouple 1, 2 and 3.

**Table 1:** Descriptive statistics (median and interquartile range) of the absolute mean temperatures (°C) over a measurement in thermocouple 1, located in between the MWA antenna and the liver surface, thermocouple 2, located at 5 mm from the liver surface in the adjacent tissue and thermocouple 3 located at 15 mm of the surface in the adjacent tissue.

		Median (°C)	Interquartile range (°C)
Thermocouple 1	Saline (*n* = 20)	54.60	49.28 – 62.72
	D5W *(n=20)*	57.46	48.89 – 63.80
Thermocouple 2	Saline *(n=20)*	18.52	17.87 – 18.83
	D5W *(n=20)*	18.06	17.63 – 18.79
Thermocouple 3	Saline *(n=20)*	17.44	16.82 – 17.59
	D5W *(n=20)*	17.40	16.89 – 17.89

At a hydrodissection fluid thickness of 1 mm, the insulating effect of D5W was better than that of saline: average temperature increase 4.6 °C and 6.8 °C, respectively, measured with thermocouple 2 (Appendix A). For hydrodissection fluid thicknesses ≥ 8 mm, there was no difference observed in temperature increase between the hydrodissection fluids. Using a hydrodissection fluid thickness of 10 mm, the temperature increase was < 1.0 °C for both D5W and saline. At 15 mm within the adjacent tissue the temperature increase was < 1.0 °C in 38 out of 40 (95 %) of the experiments, regardless of the hydrodissection fluid thickness.

**Fig. 2:**
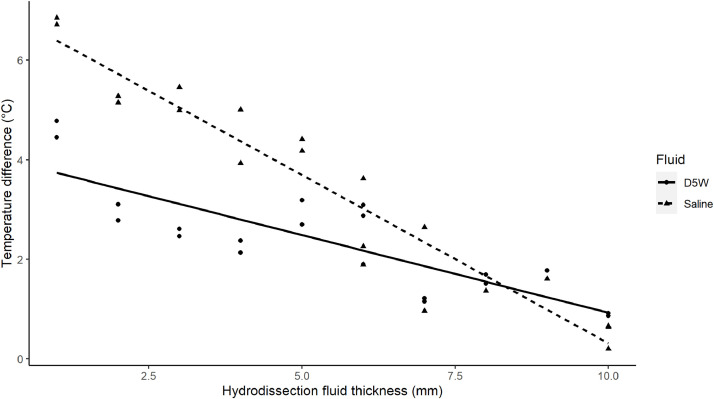
Maximum temperature increase (in degrees Celsius) measured in thermocouple 2, located at 5 mm from the surface of the adjacent porcine liver tissue, for different hydrodissection fluid thicknesses (mm) and both fluids, i.e., 5 % dextrose in (D5W) and 0.9 % NaCl solution in water (saline). A linear relationship is plotted for each fluid. Saline demonstrates a steeper slope compared to D5W. As the hydrodissection fluid thickness increases, the temperature increase measured for both fluids is no longer notable different, resulting in intersection of the lines.

Fig. 2 displays the relationship between the hydrodissection fluid thickness and the temperature increase measured in thermocouple 2 for D5W and saline. A negative linear trend is observed between the hydrodissection fluid thickness and temperature increase for both hydrodissection fluids. For thermocouple 2, a negative correlation was found between the hydrodissection fluid thickness (mm) and the temperature increase (°C) for saline hydrodissection (*r*(18) = −0.96, *p* < 0.001) as well as for D5W hydrodissection (*r*(18) = −0.81, *p* < 0.001). These correlations coefficients differed significantly (*p* = 0.011).

## 4 Discussion

This ex vivo experimental study compares the effectiveness of hydrodissection using saline or D5W during MWA at different hydrodissection fluid thicknesses. At 5 mm from the surface of adjacent tissue, the effectiveness of hydrodissection using D5W was significantly less dependent on hydrodissection fluid thickness compared to saline. This implies that D5W may be a better insulator and may prevent adjacent critical structures better from heating than saline.

It is well known that D5W is a better insulator in RFA, but this is, to our knowledge, the first experimental research to compare hydrodissection fluids in MWA [[5], [6], [7], [8]]. In our study, D5W provided better insulation than saline and therefore could be considered as first choice hydrodissection fluid in MWA. For hydrodissection fluid thicknesses ≥ 8 mm, the physical barrier was probably sufficient to compensate for the insulation difference, since a temperature increase of < 2 °C in the adjacent tissue was observed for both D5W and saline. This indicates that saline can safely be used if sufficient hydrodissection fluid thickness can be achieved. A reason to prefer saline could be that it is better tolerated by the body for high volume hydrodissection. The safe use of hydrodissection using saline in clinical practice has been reported for hydrodissection fluid thicknesses > 5 mm during MWA [[7], [8], [9], [10], [11], [12]]. However, sufficient hydrodissection fluid thicknesses (> 5 mm) might not be warranted during the entire ablation procedure, as the fluid may disperse away from its intended site. This may be less of an issue when using D5W, as suggested by findings of this study, giving the interventional radiologist more confidence to radically ablate the lesion. To address the challenge of dispersion in clinical practice, continuous fluid infusion could be used to ensure sufficient hydrodissection distances. It should be noted that this study did not involve experiments using continuous fluid flushing.

Different factors determine whether MWA can be safely performed with hydrodissection. Firstly, the distance from the MWA antenna to the capsule of the target organ. If the antenna is close to the hydrodissection fluid, there may potentially be direct heating of the fluid by the microwaves. Secondly, the ablation settings, i.e., power and time, are a factor to take into consideration. Higher power and/or longer ablation times result in higher temperatures and thus increase the risk of thermal injury to adjacent structures. Other important factors are the thermal properties of the hydrodissection fluid and hydrodissection fluid thickness. There are different modes of heat transfer involved in hydrodissection: thermal conduction, free convection, and direct heating. The interaction between these mechanisms is complex. Using liquid hydrodissection fluids, thermal convection emerged as the dominant heat transfer process [13]. The thermal convection rate is dependent on the viscosity of the fluids. Since D5W has a higher viscosity then saline, the convection rate of D5W is lower [14]. Therefore, heat dissipates less in D5W compared to saline resulting in a better insulating capacity of D5W. Fluids with a better insulating capacity offer the advantage that a smaller hydrodissection fluid thickness would be needed to prevent unintended thermal injury to adjacent structures. Our study findings are consistent with this as D5W shows lower temperature increases compared to saline at small hydrodissection fluid thicknesses and is less dependent on the fluid thickness (*r*(18) = −0.81 for D5W versus *r*(18) = −0.96 for saline). Potentially, fluid with higher dextrose concentrations may offer even better insulation, but this was not investigated in our study.

In clinical practice, contrast-enhanced hydrodissection fluid is frequently used to enhance its visibility on imaging and improve the differentiation of the fluid from the adjacent organs [15]. The viscosity of iodinated contrast medium, the most commonly used contrast agent, depends on its concentration and temperature, but generally has a higher viscosity than saline and D5W [16]. Consequently, the addition of contrast agent to either of the fluids results in an increase in viscosity. The addition of contrast agent, therefore, may diminish the observed difference between the two fluids. However, further research is needed to study the extent of this effect.

The most important limitation of this study is the ex vivo design using explanted porcine livers. Heat distribution is likely to be different in a living, perfused organ. Moreover, the experiments were performed at room temperature which differs from body temperatures. This might result in an underestimation of the measured temperature increase in the adjacent tissue, since thermal conductivity of tissue is temperature dependent [17,18].

Furthermore, porcine livers served as the adjacent tissue in the ex vivo set-up, while the thermal and electrical conductivity of possible adjacent tissues, i.e., diaphragm and gastrointestinal tract will vary from that of the liver [18]. Additionally, in clinical practice, the absolute maximum temperature is most relevant for evaluating the risk of thermal damage. In the ex vivo experiments a relatively small maximum temperature increase of 6 °C is observed at the smallest hydrodissection distance of 1 mm. However, these measurements are not directly transferable to clinical practice due to the aforementioned limitations, the limited number of measurements per hydrodissection fluid thickness and the relatively short ablation time. Moreover, limitations can be identified in the design of the experiments. Despite efforts to standardize antenna and thermocouple positions, minor variations in positions between the experiments could have occurred.

## 5 Conclusion

According to the results of this ex vivo experimental study, the effectiveness of hydrodissection using D5W is significantly less dependent on hydrodissection fluid thickness compared to saline. This suggests that D5W may be a better insulator and may be more effective in protecting adjacent critical structures from heating during MWA.

## Funding sources

This research did not receive any specific grant from funding agencies in the public, commercial, or not-for-profit sectors.

## CRediT authorship contribution statement

**Gonnie C.M. van Erp:** Conceptualization, Methodology, Formal analysis, Investigation, Writing – original draft, Writing – review & editing, Resources. **Pim Hendriks:** Conceptualization, Methodology, Writing – review & editing, Resources, Supervision. **Sophie A. van den Hurk:** Methodology, Formal analysis, Investigation, Writing – review & editing, Resources. **Hannah F. Winder:** Methodology, Formal analysis, Investigation, Writing – review & editing, Resources. **Willemijn P.M. Scholtes:** Methodology, Formal analysis, Investigation, Writing – review & editing, Resources. **Lara B.E.M. De Bats:** Methodology, Formal analysis, Investigation, Writing – review & editing, Resources. **Jouke Dijkstra:** Writing – review & editing, Supervision. **Mark C. Burgmans:** Conceptualization, Methodology, Writing – review & editing, Resources, Supervision.

## Declaration of competing interest

The authors report there are no competing interests to declare.

## Appendix Supplementary materials

Image, application 1
