# Supplementary material for: Hydrodissection in microwave ablation: the effectiveness of 0.9 % NaCl versus 5 % dextrose in an ex vivo experimental set-up
Source: Res Diagn Interv Imaging. 2025 May 20;14:100060. doi: 10.1016/j.redii.2025.100060 (PMC12148655; doi:10.1016/j.redii.2025.100060)
Supplement: Supplementary file 1 [file mmc1.docx]

## Appendix A: Minimum temperature, maximum temperature and temperature increase in thermocouple 2 and 3 for all experiments

| Hydrodissection fluid thickness | Hydrodissection fluid | Thermocouple 2 | | | Thermocouple 3 | | | |
| --- | --- | --- | --- | --- | --- | --- | --- | --- |
|  |  | Minimum temperature (°C) | Maximum temperature (°C) | Temperature increase (°C) | Minimum temperature (°C) | Maximum temperature (°C) | Temperature increase (°C) |  |
| 1 mm | Saline | 16.97 | 23.82 | 6.85 | 17.30 | 17.90 | 0.60 |  |
|  | Saline | 16.33 | 23.05 | 6.72 | 16.51 | 16.92 | 0.41 |  |
|  | D5W | 17.85 | 22.30 | 4.78 | 16.45 | 17.23 | 0.77 |  |
|  | D5W | 16.23 | 21.01 | 4.45 | 17.80 | 18.00 | 0.20 |  |
| 2 mm | Saline | 17.50 | 22.65 | 5.15 | 17.25 | 17.97 | 0.72 |  |
|  | Saline | 15.87 | 21.15 | 5.28 | 15.58 | 16.57 | 0.99 |  |
|  | D5W | 19.09 | 21.87 | 3.10 | 17.14 | 17.95 | 0.81 |  |
|  | D5W | 17.01 | 20.11 | 2.78 | 19.11 | 20.17 | 1.06 |  |
| 3 mm | Saline | 17.24 | 22.23 | 5.00 | 17.30 | 17.63 | 0.33 |  |
|  | Saline | 16.95 | 22.40 | 5.46 | 17.05 | 17.72 | 0.67 |  |
|  | D5W | 16.94 | 19.41 | 2.61 | 16.40 | 16.93 | 0.53 |  |
|  | D5W | 16.35 | 18.96 | 2.46 | 16.85 | 17.37 | 0.52 |  |
| 4 mm | Saline | 17.06 | 22.07 | 5.01 | 16.25 | 17.18 | 0.93 |  |
|  | Saline | 17.43 | 21.35 | 3.93 | 17.24 | 17.88 | 0.64 |  |
|  | D5W | 17.32 | 19.45 | 2.14 | 17.22 | 17.70 | 0.48 |  |
|  | D5W | 17.79 | 20.17 | 2.38 | 17.36 | 17.69 | 0.33 |  |
| 5 mm | Saline | 17.27 | 21.44 | 4.18 | 17.25 | 17.78 | 0.53 |  |
|  | Saline | 16.80 | 21.21 | 4.41 | 16.85 | 17.25 | 0.39 |  |
|  | D5W | 17.34 | 20.52 | 3.19 | 17.38 | 17.92 | 0.54 |  |
|  | D5W | 17.08 | 19.78 | 2.70 | 17.03 | 17.95 | 0.92 |  |
| 6 mm | Saline | 17.27 | 20.90 | 3.62 | 17.59 | 17.74 | 0.15 |  |
|  | Saline | 16.30 | 18.56 | 2.26 | 16.29 | 17.04 | 0.75 |  |
|  | Saline | 16.39 | 18.27 | 1.89 | 16.51 | 17.49 | 0.99 |  |
|  | D5W | 17.76 | 19.66 | 1.90 | 14.18 | 15.40 | 1.22 |  |
|  | D5W | 15.33 | 18.20 | 2.88 | 17.76 | 18.06 | 0.30 |  |
|  | D5W | 18.35 | 21.44 | 3.09 | 18.33 | 18.99 | 0.66 |  |
| 7 mm | Saline | 17.00 | 17.92 | 0.96 | 17.17 | 17.41 | 0.24 |  |
|  | Saline | 17.83 | 20.47 | 2.64 | 17.33 | 17.83 | 0.50 |  |
|  | D5W | 17.63 | 18.79 | 1.15 | 17.68 | 18.36 | 0.69 |  |
|  | D5W | 16.01 | 17.22 | 1.22 | 15.96 | 16.79 | 0.83 |  |
| 8 mm | Saline | 16.01 | 17.38 | 1.36 | 14.75 | 15.60 | 0.85 |  |
|  | D5W | 16.87 | 18.38 | 1.51 | 16.71 | 17.22 | 0.52 |  |
|  | D5W | 18.74 | 20.43 | 1.69 | 18.98 | 19.23 | 0.30 |  |
| 9 mm | Saline | 18.00 | 19.61 | 1.61 | 18.07 | 18.41 | 0.35 |  |
|  | D5W | 17.46 | 19.23 | 1.78 | 17.04 | 17.71 | 0.67 |  |
| 10 mm | Saline | 17.99 | 18.19 | 0.20 | 18.11 | 18.27 | 0.16 |  |
|  | Saline | 18.56 | 19.20 | 0.64 | 18.65 | 19.04 | 0.39 |  |
|  | Saline | 18.32 | 18.98 | 0.66 | 18.01 | 18.20 | 0.20 |  |
|  | D5W | 19.11 | 19.97 | 0.86 | 19.25 | 19.40 | 0.15 |  |
|  | D5W | 16.49 | 17.41 | 0.92 | 16.29 | 16.59 | 0.30 |  |
